# Self-Reported Systemic Sclerosis-Related Symptoms Are More Prevalent in Subjects with Raynaud’s Phenomenon in the Lifelines Population: Focus on Pulmonary Complications

**DOI:** 10.3390/diagnostics13132160

**Published:** 2023-06-25

**Authors:** Saskia Corine van de Zande, Amaal Eman Abdulle, Yehya Al-Adwi, Alja Stel, Karina de Leeuw, Elisabeth Brouwer, Suzanne Arends, Christiaan Tji Gan, Harry van Goor, Douwe Johannes Mulder

**Affiliations:** 1 Department of Internal Medicine, Division of Vascular Medicine, University Medical Centre Groningen, University of Groningen, 9712 Groningen, The Netherlands; a.eman.abdulle@umcg.nl (A.E.A.); y.a.s.mohammed@umcg.nl (Y.A.-A.); d.j.mulder@umcg.nl (D.J.M.); 2 Department of Rheumatology and Clinical Immunology, University Medical Centre Groningen, University of Groningen, 9712 Groningen, The Netherlands; a.j.stel@umcg.nl (A.S.); k.de.leeuw@umcg.nl (K.d.L.); e.brouwer@umcg.nl (E.B.); s.arends@umcg.nl (S.A.); 3 Department of Pulmonary Diseases and Tuberculosis, University Medical Centre Groningen, University of Groningen, 9712 Groningen, The Netherlands; c.t.gan@umcg.nl; 4 Department of Pathology and Medical Biology, University Medical Centre Groningen, University of Groningen, 9712 Groningen, The Netherlands; h.van.goor@umcg.nl

**Keywords:** systemic sclerosis, Raynaud’s phenomenon, epidemiology, pulmonary involvement, skin autofluorescence

## Abstract

Puffy fingers and Raynaud’s phenomenon (RP) are important clinical predictors of the development of systemic sclerosis (SSc). We aim to assess the prevalence of SSc-related symptoms, explore pulmonary symptoms, and test the usefulness of skin autofluorescence (SAF) as a non-invasive marker for Advanced Glycation Endproducts (AGEs). Subjects from the Lifelines Cohort Study with known connective tissue disease (CTD) were excluded. Patient characteristics, SAF, self-reported pulmonary symptoms, and spirometry were obtained. Subjects (*n* = 73,948) were categorized into definite RP (5.3%) with and without SSc-related symptoms and non-RP. Prevalence of at least one potential SSc-related symptom (other than RP) was 8.7%; 23.5% in subjects with RP and 7.1% without RP (*p* < 0.001). Subjects with RP and additional SSc-related symptoms more frequently reported dyspnea at rest, dyspnea after exertion, and self-reported pulmonary fibrosis, and had the lowest mean forced vital capacity compared to the other groups (RP without SSc-related symptoms and no RP, both *p* < 0.001). In multivariate regression, dyspnea at rest/on exertion remained associated with an increased risk of SSc-related symptoms in subjects with RP (both *p* < 0.001). SAF was higher in subjects with RP and SSc-related symptoms compared to the other groups (*p* < 0.001), but this difference was not significant after correction for potential confounders. The prevalence of SSc-related symptoms was approximately three-fold higher in subjects with RP. Pulmonary symptoms are more prevalent in subjects with RP who also reported additional potential SSc-related symptoms. This might suggest that (suspected) early SSc develops more insidiously than acknowledged. According to this study, SAF is no marker for early detection of SSc.

## 1. Introduction

Despite many efforts, the exact underlying pathology of systemic sclerosis (SSc) remains unknown, and effective disease-modifying treatment is currently unavailable [1,2]. Although some studies have reported that prevalence rates of SSc range from 30 to 300 cases per million individuals [3,4,5,6], precise estimates on earlier stages of the disease are missing. This could be explained by the fact that the previously used criteria for SSc, namely the 1980 American College of Rheumatology criteria, were shown to have a low sensitivity for the detection of early SSc [7,8,9,10]. Although more recently proposed criteria for very early diagnosis of SSc (VEDOSS) have greatly improved the diagnosis of patients with early SSc [11], no studies exist that have systematically assessed the occurrence of SSc-related symptoms in the general population.

It was previously reported that the presence of Raynaud’s phenomenon (RP), puffy fingers, and positive Antinuclear Antibody (ANA) should be considered important predictors of an underlying SSc [12]. A previously conducted study demonstrated that patients with RP, abnormal capillaroscopic pattern, and SSc-specific antibodies had a 79.5% probability of developing definite SSc after 9 years of follow-up [13]. However, the predictive value of the different SSc-related symptoms in the general population remains unknown. Knowing the natural course of early disease would facilitate strategies for the detection of early disease, early organ involvement (including pulmonary involvement), and may even allow the development of early, potentially disease-modifying strategies [14].

Pulmonary involvement, consisting of pulmonary arterial hypertension (PAH) and interstitial lung disease (ILD), is the leading cause of death in patients with SSc [15]. Hemodynamically proven rates of PAH vary between 5 and 12% [16,17,18,19]. ILD is estimated to affect approximately 53% of the patients with diffuse cutaneous SSc, and 35% of the patients with limited cutaneous SSc [20]. At the start of pulmonary involvement, the symptoms may develop more insidiously, with limited symptoms (such as dyspnea on exertion), or even symptom-free [21]. However, the detrimental outcome of both ILD and PAH further underlines the need for early screening methods. Therefore, to optimally use “the window of opportunity”, more focus on the early identification of SSc and especially pulmonary involvement is needed.

Higher oxidative stress levels are associated with pulmonary involvement in SSc [22,23,24]. Oxidative stress is related to the formation of Advanced Glycation Endproducts (AGEs) [25], which can be easily measured non-invasively as skin autofluorescence (SAF) using the AGE Reader on a large-scale basis. Earlier studies report conflicting results of an increase of AGEs in SSc patients compared with healthy controls [26,27,28,29,30].

We aimed to assess the prevalence of SSc-related symptoms and SAF levels in the general population and in subjects with RP, without a known history of connective tissue disease (CTD), therefore attempting to estimate those at risk of developing or already suffering from undiagnosed SSc. Second, we focused on the pulmonary involvement (based on self-reported symptoms and pulmonary function tests) in subjects with self-reported RP and the possible association with SSc-related symptoms. Thirdly, we explored the role of SAF as a potential early biomarker for pulmonary involvement in patients with RP.

## 2. Materials and Methods

We analyzed data from the Lifelines Cohort Study. Lifelines is a multi-disciplinary prospective population-based cohort study examining in a unique three-generation design the health and health-related behaviors of 167,729 persons living in the North of the Netherlands. It employs a broad range of investigative procedures in assessing the biomedical, socio-demographic, behavioral, physical, and psychological factors which contribute to the health and disease of the general population, with a special focus on multi-morbidity and complex genetics [31]. Before the first visit to the Lifelines outpatient clinic, all subjects were asked to complete several self-administered questionnaires, including the CTD screening questionnaire. In addition, these subjects provided (self-reported) information about their medical history, current diseases, use of medication, and health behavior. The Lifelines protocol was approved by the UMCG Medical ethical committee under number 2007/152 and conducted in accordance with the Declaration of Helsinki. All subjects provided written informed consent before enrolment.

### 2.1. Subjects

For the present analysis, subjects were ≥18 years old. Subjects were excluded with self-reported underlying CTD including systemic lupus erythematosus, primary Sjögren syndrome, and systemic sclerosis (self-reported), or if the CTD screening questionnaire was missing. In accordance with the terms of Lifelines, numbers <10 were not reported to increase privacy (and decrease traceability of the participant).

### 2.2. Clinical Examination and Investigations

During the first visit to the Lifelines outpatient clinic, all subjects underwent a general clinical examination, followed by blood and urine collection. Body mass index (BMI) was calculated. Pulmonary function was measured with spirometry (Welch Allyn device version 1.6.0.489) according to standard operating procedures. The forced vital capacity (FVC) was used as a measure of lung function. Predicted FVC was calculated according to the following equations [32]:

$${\mathrm{FVC}}_{\mathrm{men}}=\left(0.0576\times \mathrm{Height}\right)-\left(0.0269\times \mathrm{Age}\right)-4.34$$

$${\mathrm{FVC}}_{\mathrm{women}}=\left(0.0443\times \mathrm{Height}\right)-\left(0.026\times \mathrm{Age}\right)-2.89$$

FVC values were dichotomized as FVC < 70% or FVC ≥ 70% of the predicted value.

SAF measurements were performed with the AGE Reader (DiagnOptics Technologies BV, Groningen, The Netherlands). During the first visit, SAF measurements were performed by trained staff on the volar site of the right and left forearm (10 cm below the elbow). Reference values and a detailed description of the AGE Reader have been published before [33,34]. In short, a skin surface of approximately 4 cm^2^ is illuminated by the AGE Reader and guarded against surrounding light. The excitation light source has a wavelength between 300 nm and 420 nm, with a peak intensity of 370 nm. The emission light and reflected excitation light from the skin are measured by an internal spectrometer in the range of 300–600 nm. The average emitted light intensity per nm (range 420 to 600 nm) is divided by the average excitation light intensity per nm (range 300 to 420 nm) and multiplied by 100 to calculate SAF (expressed in arbitrary units (AU)). A previous study found that when repeated SAF measurements were taken over one day the error was approximately 5% [33]. Furthermore, per year of ageing there is a mean linear increase of 0.023 AU [34].

### 2.3. Patient Characteristics and Self-Reported Symptoms

For the current study, the following patient characteristics were obtained: gender, age, smoking behavior (e.g., current or previous smoking), height (cm), weight (kg), presence of comorbidities (i.e., arrhythmias, heart failure, myocardial infarction, stroke, diabetes, hypercholesterolemia, hypertension, cancer, and COPD), and demographic variables. Use of medication (i.e., anticoagulants, adrenergic and dopaminergic agents, antihypertensive agents, immunosuppressive drugs, central acting sympathomimetic, anti-cancer treatment, and beta-blocking agents) was verified at baseline using the Anatomical Therapeutic Chemical (ATC) Classification System. Patients were asked to fill in a questionnaire regarding pulmonary symptoms, including self-reported dyspnea at rest and on exertion (present/not present), as well as self-reported pulmonary fibrosis.

The validated CTD screening questionnaire, which comprised 30 questions, was used to identify RP and SSc-related symptoms during the follow-up assessment [35,36], which had taken place within 3 years following the baseline assessment (median 25 months [IQR 23–31]). This questionnaire was translated from English to Dutch by a two-panel methodology, detailed information can be found in our previous work [37]. Subjects who stated experiencing unusual sensitivity to cold and reported bi- or triphasic discoloration of the fingers were classified as definite RP [38]. The presence of the other SSc-related symptoms was obtained based on the following questions: “*Have you ever had puffy, swollen fingers for more than a month?*” (question 1), “*Have you ever had skin thickening or tightening of the face, neck, trunk, arms, or legs (not caused by fluid retention)?*” (question 2), “*Have you ever had skin thickening of the fingers or toes?*” (question 3), and “*Have you ever had sores leaving scars in the finger tips?*” (question 4). For the current study, three groups were formed, namely: (1) subjects without RP (*n* = 73,948), (2) subjects with RP but without SSc-related symptoms (*n* = 2993), and (3) subjects with RP and with SSc-related symptoms (*n* = 918).

### 2.4. Statistical Analysis

Statistics were performed with The Statistical Product and Service Solutions (SPSS; version 22, Released 2013, IBM Corp., Armonk, NY, USA). Results were expressed as numbers of subjects (percentages), mean ± SD, or median (IQR) for categorical, normally, and non-normally distributed data, respectively. Chi-Square test and one-way ANOVA were used as appropriate to compare characteristics between three groups: subjects with RP with SSc-related symptoms, subjects with RP without SSc-related symptoms, and subjects without RP. Chi-Square test and independent samples t-test were used to compare characteristics between subjects with RP with and without SSc-related symptoms. In subjects with RP, binary logistic regression (presented as odds ratio and 95% CI) was performed with SSc-related symptoms (present vs. absent) as the dependent variable to assess the association of signs of pulmonary involvement (i.e., self-reported symptoms and FVC measurements) or SAF levels with the presence of SSc-related symptoms. Variables that were statistically significant in the univariate analyses were further analyzed in multivariate logistic regression (enter method) to correct for potential confounders (i.e., age, gender, BMI, current smoking, self-reported COPD, self-reported heart failure for pulmonary involvement and age, gender, BMI, current smoking, diabetes, hypertension, hypercholesterolemia, and heart failure for SAF levels). To avoid the issue of multicollinearity caused by the overlapping of pulmonary problems (i.e., dyspnea at rest, dyspnea on exertion, and FVC) which mainly occur simultaneously, we analyzed them in separate models. *p*-values below 0.05 were considered statistically significant.

## 3. Results

### 3.1. Patient Characteristics

A total of 73,948 subjects were included (non-RP *n* = 70,037 (94.7%), definite RP *n* = 3911 (5.3%)). The non-RP group had a mean age of 45.7 years (SD ± 12.8), 58.7% were women, and the RP group had a mean age of 42.4 years (SD ± 13.0) and 80.3% were women. Other patient characteristics are described in Table 1.

### 3.2. Prevalence of SSc-Related Symptoms in Patients with and without Raynaud’s Phenomenon

**Figure 1:**
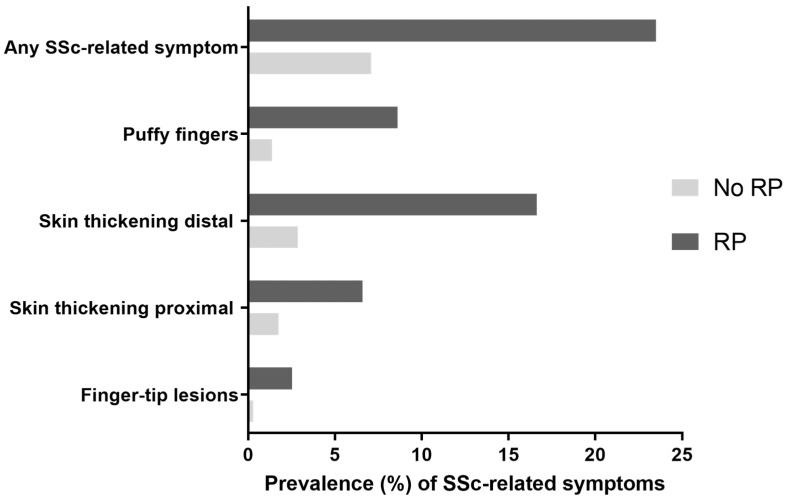
Prevalence of SSc-related symptoms in the non-RP group and RP group. The presence of SSc-related symptoms was based on the four questions mentioned in part 2.3 of the Methods section. Question 1 is mentioned as ‘puffy fingers’, question 2 as “skin thickening distal”, question 3 as “skin thickening proximal” and question 4 as “finger-tip lesions”. SSc = Systemic Sclerosis and RP = Raynaud’s Phenomenon.

In the total cohort, a prevalence of 8.7% [95% CI 8.5–8.9] of at least one SSc-related symptom (other than RP) was observed. Additionally, a prevalence of 7.1% [95% CI 5.9–6.2] of any SSc-related symptoms was observed in the non-RP group compared to 23.5% [95% CI 22.2–24.8] in the RP group (*p* < 0.001). Regarding these SSc-related symptoms, 1.4% of the subjects in the non-RP group reported having experienced puffy fingers, as compared to 8.6% of the subjects with RP (*p* < 0.001, Figure 1). Skin thickening of the fingers or toes was reported in 2.9% of the subjects in the non-RP group, compared to 16.6% of the subjects with RP (*p* < 0.001). In addition, skin thickening of the face, neck, trunk, arms, or legs was reported in 1.8% of the subjects in the non-RP group, and 6.6% of the participant with RP (*p* < 0.001). Finger-tip lesions were reported in 0.3% of the subjects in the non-RP group, compared to 2.5% of the subjects with RP (*p* < 0.001).

### 3.3. Differences in Patient Characteristics between the Groups

Subjects with RP and self-reported SSc-related symptoms were significantly older compared to subjects with RP without SSc-related symptoms (*p* < 0.001, Table 1). In addition, comorbidities not directly associated with SSc were significantly more prevalent in subjects with RP and SSc-related symptoms as compared to the other groups; however, the multivariate analysis revealed that these comorbidities were not associated with an increased risk of SSc-related symptoms (data not shown).

### 3.4. Analysis of Skin Autofluorescence and Its Relation to SSc-Related Symptoms

Subjects with RP and SSc-related symptoms showed a higher SAF compared to the other groups (all *p* < 0.001). Univariate analysis showed that SAF was significantly associated with SSc-related complaints. In multivariate analysis, this association was no longer statistically significant after correcting for known potential confounders, including age, gender, BMI, self-reported heart failure, current smoking, diabetes, hypertension, hypercholesterolemia, and heart failure (Table 2).

### 3.5. Exploratory Analysis of Pulmonary Symptoms in Subjects with Raynaud’s Phenomenon, and Its Relation to SSc-Related Symptoms

Subjects with RP and SSc-related symptoms more frequently reported dyspnea at rest, dyspnea on exertion, and self-reported pulmonary fibrosis compared to the other groups (all *p* < 0.001). In addition, subjects with RP and SSc-related symptoms had the lowest FVC mean (*p* = 0.009) (Table 1). Univariate analysis showed that FVC, dyspnea at rest, and dyspnea on exertion were significantly associated with the risk of SSc-related complaints. In multivariate analysis, dyspnea at rest and dyspnea on exertion remained significantly associated with an increased risk of SSc-related symptoms in subjects with RP after correction for known potential confounders (Table 3).

## 4. Discussion

In this large cohort of individuals from the general population without a known history of CTD, we found a three-fold higher prevalence of self-reported potential SSc-related symptoms in subjects with RP compared to those without RP. In addition, pulmonary symptoms, as a putative proxy of early pulmonary involvement, were more prevalent in subjects with self-reported RP and SSc-related symptoms.

Early SSc is often characterized by at least two of the following signs: the presence of RP, puffy fingers, typical capillaroscopic changes, and SSc-specific antibodies. These signs form the bases of the internationally recognized criteria for “very early diagnosis of SSc” (VEDOSS) [11,39]. Although several studies have underlined the importance of epidemiological studies on early SSc, the current study is the first to investigate the prevalence of SSc-related symptoms in the general population. Spencer-Green (1998) previously reported that 12.6% of the patients with primary RP eventually developed CTDs [40]. Therefore, subjects with RP and SSc-related symptoms in the current cohort might even have a higher lifetime risk of developing a CTD. This higher risk is because they experience at least two SSc-related symptoms (i.e., RP and at least one other SSc-related symptom). However, we must underline the fact that a substantial number of subjects without RP also reported SSc-related symptoms. On the other hand, it seems unlikely that these subjects without RP will ever develop SSc. This is in line with the study of Schneeberger et al. (2013) who was only able to identify a small sub-group of SSc patients (3.7%) without RP in the EUSTAR database [41]. In addition, we observed that subjects with RP, but without SSc-related symptoms were significantly younger than subjects with RP and with SSc-related symptoms. This might be because SSc often occurs in middle-aged patients. The high prevalence of SSc-related symptoms in subjects without RP can likely be explained by the self-reported nature of these questions. For instance, it was previously demonstrated that self-reported questionnaires are more sensitive to bias (e.g., desirability bias, response bias, acquiescent bias) [42]. Despite these known shortcomings of self-reported questionnaires, the CTD screening questionnaire is a validated method, which has been designed to identify several CTDs in the general population [36].

Although it has been reported that the presence of RP, puffy fingers, and positive ANA should be considered important predictive signs of an underlying SSc, the predictive value of the different SSc-related symptoms examined in our study (in combination with RP), in the general population remains unknown [12]. The current study found that distal skin thickening was the most frequently reported SSc-related complaint in subjects with RP. However, this symptom was also frequently reported in subjects without RP, indicating that skin thickening might not be the best distinguishing (early) symptom. For instance, several other diseases (e.g., peripheral edema, endocrinopathies, nephrogenic fibrosing dermopathy) can also present with or give the patient the perception of thickening of the skin (both distal and proximal), and may therefore mimic SSc [43]. The current study found a prevalence of 8.6% of puffy fingers in subjects with RP, compared to only 1.4% in the subjects without RP. According to many scleroderma experts who participated in the Delphi exercise, the occurrence of puffy fingers is one of three red flags (alongside RP and positive ANA) that should raise concerns about very early SSc [12]. Although the negative predictive value of puffy fingers remains unknown due to the absence of longitudinal observations, the positive predictive value of the progression to SSc in patients with RP, positive ANA test, and puffy fingers was found to be 88.5%, compared to 33.9% in subjects without puffy fingers [11,44]. Despite the significant value of puffy fingers in the diagnosis of SSc [12], subjects with RP and SSc-related symptoms in the current study had a significantly higher BMI as compared to subjects with RP but without SSc-related symptoms. Therefore, subjects with RP and SSc-related symptoms might misinterpret the thicker fingers (due to a high BMI) for puffy fingers. However, subjects in the non-RP group had the highest BMI as compared to the other groups, and these subjects had a much lower prevalence rate of puffy fingers.

Although information on the predictive value of finger-tip lesions remains unknown, the current study found that finger-tip lesions were the least reported SSc-related symptom. The low prevalence of finger-tip lesions, as compared to the other SSc-related symptoms, may reflect the relatively good state of the microcirculation of these subjects [45]. Moreover, this might also suggest that finger-tip lesions occur in a later stage of the disease. However, prospective studies are needed to elucidate the predictive value of all the SSc-related symptoms in subjects with RP from the general population.

In our exploratory sub-study, we aimed to determine the prevalence of pulmonary symptoms in subjects with RP and SSc-related symptoms. The current study showed that pulmonary symptoms, as well as lower FVC volumes, were more prevalent in subjects with RP and SSc-related symptoms. It is important to mention that pulmonary complaints can have multiple etiologies in the general population. However, we have demonstrated that dyspnea at rest and dyspnea on exertion were associated with an increased risk of SSc-related symptoms in subjects with RP, independent of known confounders (e.g., smoking, COPD, and heart failure). Although studies reporting on the prevalence of preclinical organ involvement in subjects with pre-SSc or VEDOSS are very limited, Valentini et al. (2012) reported that preclinical lung abnormalities are common in patients with early SSc [39]. In addition, Aalbers et al. (1989) previously reported that 7% of the patients with non-secondary RP, and 60% of the patients with secondary RP (without already an underlying CTD) had a decreased pulmonary diffusing capacity [46]. These studies indicate that pulmonary involvement might be prevalent in both early SSc and in subjects with secondary RP, and the current study may provide new insights into the prevalence of pulmonary complaints in subjects at risk of developing SSc. Additionally, it may also imply that a subset of these subjects may already suffer from undiagnosed SSc, who have not sought medical advice and might be at substantial risk of severe pulmonary complications. As a result, we highly encourage further diagnostic work-up in patients who present with RP, SSc-related symptoms, and pulmonary symptoms.

Previous research has shown that there is a correlation between oxidative stress (8-iso-PGF_2α_) levels (a urinary oxidative stress marker) and pulmonary involvement. The level of 8-iso-PGF_2α_ is higher in SSc patients with lung involvement [47]. Furthermore, during fibrotic processes, oxidative stress plays a role in SSc [48,49]. SAF—as a validated non-invasive marker for oxidative stress—was significantly higher in the group with RP and SSc-related symptoms. However, after correcting for confounders it did not remain significant. The subjects with RP and SSc-related symptoms were older than the subjects with RP but without SSc-related symptoms, this might explain why SAF did not remain significantly associated in the multivariate analysis. SAF is increased with a rate of 0.002 per year of ageing [34]. Another explanation might be that SAF only plays a role later in the disease. Earlier studies stated that oxidative stress plays a role in inflammation, endothelial injury, and fibrotic processes in patients with SSc [47,48,49]. On the other hand, another study found the highest levels of oxidative stress in the early stages of the disease [50]. Studies about the relation between SSc and a higher SAF level are inconclusive. Some studies found higher SAF levels in SSc patients [28,29,30], while another study showed that the SAF levels were not increased in SSc patients [27]. According to this study, SAF seems not to be a suitable marker for the early detection of SSc; however, more research needs to focus on the role of SAF after diagnosis.

The current study has some limitations. First, the self-reported nature of the questions used could lead to an overestimation of the prevalence of SSc-related symptoms. Although this could indeed be the case, the questionnaire used in this study was previously validated to screen for CTDs in large population studies. Furthermore, the self-reported RP is not confirmed by a clinician, and it cannot be concluded whether participants all comply with the criteria of having RP. However, by the question regarding biphasic and triphasic discoloration, we think that most people comply with the criteria for having RP. Second, information on nailfold capillaroscopy and ANA test are not part of the general clinical examination of the Lifelines cohort. Therefore, the exact prevalence of VEDOSS remains unknown. Future studies need to confirm whether this first line of screening will yield a substantial number of potential SSc patients as indicated by our data. Third, we do not have information on the diffusing capacity for carbon monoxide (DL_CO_) and on the total lung capacity (TLC). The latter could give us a correct estimation of restriction due to ILD, and, therefore, may help us to disentangle common pulmonary complaints from SSc-related pulmonary involvement (e.g., ILD, which is often associated with a decrease in DLco levels, and is also impaired in heart failure) [51]. Furthermore, for a large cohort, FVC is a good screening tool. FVC is not a screening instrument for PAH which is a feared late complication of SSc. DLco is more suitable, in addition to electrocardiographic changes and serum biomarkers such as nt-proBNP and uric acid. Fourth, the definition of finger-tip lesions used in the current study may have led to an overestimation of the prevalence of finger-tip lesions in the general population, as pitting scars are usually defined as pinhole-sized depressions with hyperkeratosis. However, a two-panel methodology was applied to translate the English CTD questionnaire and to ensure that the language being used in the final version would be understood by potential respondents. Fifth, given the large nature of the study, it is more likely that differences between groups are statistically significant, but not clinically relevant per se. However, the purpose of the variables in Table 1 is to indicate the differences between the three groups. Sixth, since the prevalence of SSc-related and pulmonary symptoms is relatively high, it is conceivable that other comorbidities—such as musculoskeletal and psychosocial disabilities—may have influenced these results. Furthermore, in our univariate analysis, other pulmonary diseases including COPD, or the frequency of current smokers were more prevalent in the Raynaud group with SSc-related symptoms. A future follow-up study is mandatory to exclude these factors, for example by including validated questionnaires for health-related functions. Despite these shortcomings, the current study was designed to investigate the prevalence of SSc-related symptoms in the general population, and, therefore, could help us acquire a better understanding of the epidemiology of suspected early SSc.

## 5. Conclusions

The current study demonstrated an increased prevalence of SSc-related symptoms in subjects with RP compared to those without RP, and an increased prevalence of pulmonary symptoms in those with both RP and SSc-related symptoms. Our results suggest that the prevalence of clinical features of SSc in the general population is higher than what could be expected based on the current literature. By demonstrating an increased prevalence of pulmonary symptoms (which could indicate lung involvement in patients with RP in combination with SSc-related symptoms), our study underscores the need to take these potential SSc-related symptoms seriously. SSc-related symptoms in patients with RP should always alert the patient and physician to proceed with serological, nailfold capillaroscopy investigations and lung functions tests including DLco, therefore allowing for prompt diagnosis, monitoring, and treatment of a life-threatening disease.

## Figures and Tables

**Table 1:** Patient characteristics of subjects with RP and with SSc-related symptoms, subjects with RP and without SSc-related symptoms, and subjects without RP and without SSc-related symptoms.

	RP + SSc-Related Symptoms *n* = 918	RP + No SSc-Related Symptoms *n* = 2993	^±^ *p*-Value	Non-RP Group *n* = 70,037	^×^ *p*-Value
Gender, female, *n* (%)	791 (86.2%)	2348 (78.4%)	<0.001	39,185 (55.9%)	<0.001
Age in years, mean ±SD	45 ±13	42 ±13	<0.001	45 ± 13	<0.001
Age groups, *n* (%)			<0.001		<0.001
<20 years	18 (2.0%)	89 (3.0%)		976 (1.4%)	
20–40 years	287 (31.3%)	1171 (39.1%)		21,313 (30.4%)	
40–60 years	482 (52.5%)	1445 (48.3%)		37,150 (53.0%)	
>60 years	131 (14.3%)	288 (9.6%)		10,598 (15.1%)	
BMI in kg/m^2^, mean ±SD	25 ± 5	24 ± 4	<0.001	26 ± 4	<0.001
Comorbidities, *n* (%)					
Arrhythmias	128 (13.9%)	266 (8.9%)	0.06	4842 (6.9%)	0.002
Heart failure	13 (1.4%)	21 (0.7%)	0.02	415 (0.6%)	0.001
Myocardial infarction	11 (1.2%)	21 (0.7%)	0.14	625 (0.9%)	0.33
Stroke	<10 (x%)	20 (0.7%)		417 (0.6%)	
Diabetes	29 (3.2%)	38 (1.3%)	<0.001	1600 (2.3%)	<0.001
Hypercholesterolemia	141 (15.4%)	296 (9.9%)	<0.001	9307 (13.3%)	<0.001
Hypertension	268 (29.2%)	648 (21.7%)	<0.001	14,966 (21.4%)	<0.001
Cancer (previous and current)	59 (6.4%)	140 (4.7%)	0.04	3147 (4.5%)	0.02
COPD	99 (10.8%)	152 (5.1%)	<0.001	3145 (4.5%)	<0.001
Use of medication, *n* (%)					
Anticoagulants	61 (6.6%)	101 (3.4%)	<0.001	2499 (3.6%)	<0.001
Adrenergic and dopaminergic agents	<10 (x%)	<10 (x%)		22 (0.03%)	
Antihypertensive drugs	111 (12.1%)	218 (7.3%)	<0.001	6195 (8.8%)	<0.001
Immunosuppressive drugs	50 (5.4%)	115 (3.8%)	0.03	1698 (2.4%)	<0.001
Central acting sympathomimetic	<10 (x%)	19 (0.6%)		200 (0.3%)	
Anti-cancer treatment	<10 (x%)	<10 (%)		28 (0.04%)	
Beta-blocking agents	72 (7.8%)	150 (5.0%)	0.001	3674 (5.2%)	<0.001
Smoking behavior, *n* (%)					
Current smoker	194 (21.1%)	551 (18.4%)	0.07	12,952 (18.5%)	0.14
Previous smoker	352 (38.3%)	928 (31.0%)	0.29	25,108 (35.8%)	0.04
SAF (AU) mean ± SD	(*n* = 579) 1.91 (0.46)	(*n* = 1850) 1.80 (0.45)	<0.001	(*n* = 44,484) 1.88 (0.45)	<0.001
Pulmonary function					
FVC (liter), mean ±SD	4.1 ± 0.8	4.4 ± 0.9	<0.001	4.6 ± 1.0	<0.001
FVC < 70, *n* (%)	44 (4.5%)	92 (3.1%)	0.009	3068 (4.4%)	0.003
Patient-reported dyspnea (at rest), *n* (%)	205 (22.3%)	405 (13.5%)	<0.001	5553 (7.9%)	<0.001
Patient-reported dyspnea (on an exertion), *n* (%)	465 (50.7%)	947 (31.6%)	<0.001	15,518 (22.2%)	<0.001
Patient-reported pulmonary fibrosis, *n* (%)	24 (2.6%)	30 (1.0%)	<0.001	436 (0.6%)	<0.001

^±^ *p*-value = difference between subjects with RP and SSc-related symptoms and those with RP and without SSc-related symptoms. ^×^ *p*-value = difference between the three groups.

**Table 2:** Relationship between SAF levels and SSc-related symptoms in subjects with Raynaud’s phenomenon. The multivariate analysis was corrected for age, gender, BMI, self-reported heart failure, current smoking, diabetes, hypertension, hypercholesterolemia, and heart failure.

	Univariate Analysis			Multivariate Analysis		
	B	OR [95% CI]	*p*-Value	B	OR [95% CI]	*p*-Value
SAF	0.52	1.68 [1.37–2.05]	<0.001	0.18	1.19 [0.88–1.61]	0.250

SAF = Skin Autofluorescence. B = coefficient, OR = Odds Ratio.

**Table 3:** Relationship between pulmonary symptoms and the presence of SSc-related symptoms in subjects with Raynaud’s phenomenon. The multivariate model was corrected for age, gender, BMI, self-reported heart failure, current smoking, and self-reported COPD. ^±^ not experiencing dyspnea was used as a reference.

	Univariate Analysis			Multivariate Analysis		
	B	OR [95% CI]	*p*-Value	B	OR [95% CI]	*p*-Value
FVC	−0.34	0.71 [0.64–0.80]	<0.001	−0.05	0.95 [0.80–1.13]	0.543
^±^ Dyspnea at rest	0.61	1.84 [1.53–2.22]	<0.001	0.43	1.54 [1.21–1.95]	<0.001
^±^ Dyspnea on exertion	0.79	2.20 [1.89–2.55]	<0.001	0.63	1.87 [1.56–2.25]	<0.001

FVC = Forced Vital Capacity. B = coefficient, OR = Odds Ratio.

## Data Availability

The data underlying this article will be shared on reasonable request to the corresponding author.
